# Novel insights on oral squamous cell carcinoma management using long non-coding RNAs

**DOI:** 10.32604/or.2024.052120

**Published:** 2024-09-18

**Authors:** SUBHAYAN SUR, DIMPLE DAVRAY, SOUMYA BASU, SUPRIYA KHEUR, JAYANTA KUMAR PAL, SHUCHI NAGAR, AVINASH SANAP, BHIMAPPA M. RUDAGI, SAMIR GUPTA

**Affiliations:** 1 Cancer and Translational Research Centre, Dr. D. Y. Patil Biotechnology and Bioinformatics Institute, Dr. D. Y. Patil Vidyapeeth, Tathawade, Pune, 411033, India; 2 Bioinformatics Centre, Dr. D. Y. Patil Biotechnology and Bioinformatics Institute, Dr. D. Y. Patil Vidyapeeth, Pune, 411033, India; 3 Department of Oral Pathology and Microbiology, Dr. D. Y. Patil Dental College & Hospital, Dr. D. Y. Patil Vidyapeeth, Pune, 411018, India; 4 Department of Surgical Oncology, Dr. D. Y. Patil Medical College, Hospital & Research Centre, Dr. D. Y. Patil Vidyapeeth, Pune, 411018, India

**Keywords:** Oral squamous cell carcinoma (OSCC), Head and neck cancer, Non-coding RNA, Long non-coding RNA (lncRNA), Cancer diagnosis

## Abstract

Oral squamous cell carcinoma (OSCC) is one of the most prevalent forms of head and neck squamous cell carcinomas (HNSCC) with a poor overall survival rate (about 50%), particularly in cases of metastasis. RNA-based cancer biomarkers are a relatively advanced concept, and non-coding RNAs currently have shown promising roles in the detection and treatment of various malignancies. This review underlines the function of long non-coding RNAs (lncRNAs) in the OSCC and its subsequent clinical implications. LncRNAs, a class of non-coding RNAs, are larger than 200 nucleotides and resemble mRNA in numerous ways. However, unlike mRNA, lncRNA regulates multiple druggable and non-druggable signaling molecules through simultaneous interaction with DNA, RNA, proteins, or microRNAs depending on concentration and localization in cells. Upregulation of oncogenic lncRNAs and down-regulation of tumor suppressor lncRNAs are evident in OSCC tissues and body fluids such as blood and saliva indicating their potential as valuable biomarkers. Targeted inhibition of candidate oncogenic lncRNAs or over-expression of tumor suppressor lncRNAs showed potential therapeutic roles in *in-vivo* animal models. The types of lncRNAs that are expressed differentially in OSCC tissue and bodily fluids have been systematically documented with specificity and sensitivity. This review thoroughly discusses the biological functions of such lncRNAs in OSCC cell survival, proliferation, invasion, migration, metastasis, angiogenesis, metabolism, epigenetic modification, tumor immune microenvironment, and drug resistance. Subsequently, we addressed the diagnostic and therapeutic importance of lncRNAs in OSCC pre-clinical and clinical systems, providing details on ongoing research and outlining potential future directions for advancements in this field. In essence, this review could be a valuable resource by offering comprehensive and current insights into lncRNAs in OSCC for researchers in fundamental and clinical domains.

## Introduction

Oral squamous cell carcinomas (OSCC) are one of the most common forms of head and neck squamous cell carcinomas (HNSCC) that develop in the mucosal epithelium of the oral cavity, tongue, and lips. The GLOBOCAN estimated 377,713 new cases of lip and oral cavity cancer in the world in 2020, with 177,757 deaths [1]. Males have a higher incidence and death rate [Incidence: 264,211, death: 125,022] than females [incidence: 113,502, death: 52,735] in the world. The prevalence of OSCC varies by country/region and has been linked to exposure to tobacco-derived carcinogens, excessive alcohol intake, or both. Infection with oncogenic strains of human papillomavirus (HPV) is mostly associated with tumors of the oropharynx and successful vaccination initiatives around the world may be able to prevent HPV-positive HNSCC [2]. Because of the prominence of tobacco and alcohol intake, as well as betel nut chewing, oral cavity cancer is quite common in South Central Asia (e.g., India, Sri Lanka, and Pakistan), as well as Papua New Guinea, Taiwan, and some provinces in mainland China [1,2]. According to the American Cancer Society, the incidence of oral cavity and pharyngeal cancer in the United States in 2023 was around 54,000 new cases and 11,580 fatalities [3]. Since 1991, the United States has seen a 33% decrease in total mortality, however, the annual mortality rate for oral cavity and pharyngeal cancer has increased by 2% in males and 1% in women [3].

The majority of OSCC patients require multimodality treatments and multidisciplinary care, with the exception of early-stage cancers of the oral cavity, which are typically treated with surgery. As a radiation sensitizer for patients with recurrent or metastatic cancer, the FDA has approved cetuximab, a monoclonal antibody that targets the epidermal growth factor receptor (EGFR; also known as HER1) [2]. Pembrolizumab and nivolumab, immune checkpoint inhibitors, have been licensed by the FDA to treat cisplatin-refractory recurrent or metastatic ovarian cancer [2]. The overall survival rate of OSCC is approximately 50%, and it is much lower in cases of metastasis, even with advancements in traditional therapy modalities [4]. Extensive molecular characterization and immunological profiling point to the possibility that integrating diagnostic and prognostic biomarkers into clinical practice could help get beyond challenges to targeted therapy and increase survival [2].

Numerous high-throughput sequencing-based studies and international collaborative studies like The Cancer Genome Atlas (TCGA), and International Cancer Genome Consortium (ICGC) show the importance of RNA biomarkers in cancers. For example, PAM50, a 50 mRNA gene panel has been effectively applied for molecular diagnosis of breast cancer [5] At the transcriptome level, more than 98% of the human genome is made up of non-coding RNAs such as microRNA (miRNA), long non-coding RNA (lncRNA), circular RNA (circRNA), PIWI-interacting RNA (piRNA), and small nucleolar RNAs (snoRNA) [6].

Noncoding RNAs were formerly thought to be functionless for cells but because of advancements in thorough molecular research, there has been increased interest in their role in the genesis of numerous disorders, including cancer. Among these non-coding RNAs, around 10000–16000 are long non-coding RNAs (lncRNAs), which are more than 200 nucleotides in length and are mainly transcribed by RNA polymerase II [7,8]. The lncRNAs are intergenic transcripts that overlap with other genes and are commonly capped by 7-methyl guanosine at their 5′ ends, polyadenylated at their 3′ ends, and spliced like mRNA [7,8]. LncRNAs can be found in the nucleus, the cytoplasm, or both, and depending on the localization lncRNAs regulate different cellular processes. They interact with DNA, RNAs, or proteins thereby regulating multiple events in cellular homeostasis. The ‘LncRNADisease’ database (www.cuilab.cn/lncrnadisease, accessed on 20/04/2024) enlists around 3000 lncRNAs that are associated with different diseases. Numerous lncRNAs have been shown to be dysregulated in malignancies. For instance, downregulation of the lncRNA Meg3 and dual function of the lncRNA H19, and upregulation of the lncRNAs HOTAIR and MALAT-1, have been observed in a number of cancer types, including gastric, ovarian, prostate, breast, lung, and liver cancer [9]. The coding portion of the diphtheria toxin and the lncRNA H19 promoter sequence was introduced to the BC-819 plasmid in clinical trials for bladder, ovarian, and pancreatic tumors [7]. Clinical trials have been done or initiated to evaluate diagnostic or therapeutic importance of lncRNA HOTAIR in thyroid cancer (ClinicalTrials.gov, accessed on 20/04/2024. Identifier: NCT03469544); CCAT1 in colorectal cancer (NCT04269746); LncRNA-GC1 in gastric cancer (NCT05397548, NCT05647941 and NCT05334849); MFI2-AS1 in localized clear cell kidney cancers (NCT04946266); H19 in acute lymphoblastic leukemia (NCT05943093); and lncRNAs WRAP53 and UCA-1 in hepatocellular carcinoma (NCT05088811). Inhibitors of lncMyoD (WO2015020960) and LINC01212 (US2016271163) are being used as therapeutic markers for melanoma and sarcoma treatment. These results all point to the possibility of using lncRNAs in clinical settings for cancer detection and treatment.

This review delves into the pivotal role of lncRNAs in the progression of OSCC. The findings highlight the clinical significance of these molecular players in cancer diagnosis, prognosis, and therapeutic interventions with in-depth molecular mechanisms. The review suggests that the changes in the lncRNA transcriptome hold immense potential as diagnostics and therapeutic agents, offering a deeper understanding of the relationship between lncRNA alterations and OSCC management. The concise yet comprehensive summary provides researchers in both basic science and clinical domains with invaluable, up-to-date insights into the rapidly evolving field of lncRNAs in OSCC. This resource equips them with the necessary knowledge to drive future mechanistic studies and explore the therapeutic implications of these novel discoveries, ultimately paving the way for improved patient outcomes in OSCC.

## Materials and Methods

In this review article, we have discussed the role of lncRNAs in OSCC and their clinical significance in cancer diagnosis, prognosis, and therapy. The information is obtained from research or review articles available on “PubMed,” “PubMed Central,” “Google Scholar,” “Science Direct,” and “Semantic Scholar”. LncRNA specific further functional and clinical information are obtained from https://clinicaltrials.gov/ (accessed on 20/04/2024), ‘LncRNADisease’ database (www.cuilab.cn/lncrnadisease), The Cancer Genome Atlas (TCGA) (https://www.cbioportal.org/; http://gepia.cancer-pku.cn/, accessed on 20/04/2024); PANTHER18.0 (https://pantherdb.org/, accessed on 20/04/2024); https://www.genecards.org/, accessed on 20/04/2024 and ENCORI/starBase: rnasysu.com/encori/index.php, accessed on 20/04/2024. “Long non-coding RNA” “lncRNA”, “Oral cancer”, “Oral squamous cell carcinoma (OSCC)”, “Head and neck squamous cell carcinomas (HNSCC)”, and “Head and neck cancer” are the search terms utilized. This study’s data come from studies that used pre-clinical and clinical models; it excludes information on news articles, conference proceedings, comments, and materials that are unique to a given profession.

### Current oral cancer diagnostic and prognostic markers

The OSCC is initiated by different precancerous lesions and according to the WHO categorization, it is known as an Oral Potentially Malignant Disorders (OPMD). Clinically the OPMD includes leukoplakia, erythroleukoplakia, oral submucous fibrosis, proliferative verrucous leukoplakia (PVL), erythroplakia, and dysplasia [10]. Oral dysplasia is the most advanced type of OPMD, with three severity levels: mild, moderate, and severe [10]. Medical professionals must monitor and treat patients with oral cancer and increase their chances of survival by detecting the disease early and evaluating the possible risk of an OPMD transforming malignant. Currently, the most reliable methods for diagnosing OSCC are tissue biopsy and histological analysis. Unfortunately, patients find this technique to be invasive, and painful, and causes a delay in diagnosis [11]. The early diagnosis and identification of oral pathophysiologic lesions are aided by metachromasia, which uses iodine or toluidine blue staining to stain cancerous lesions, chemiluminescence-based lumenoscopy, auto-fluorescence-based techniques such as Laser Induced Auto-fluorescence (LIAF) or Visually Enhanced Lesion scope (Velscope), optical coherence tomography (OCT), and oral brush cytology [11]. In order to facilitate a prompt assessment and treatment of oral lesions, molecular diagnostic and prognostic indicators, such as changes in DNA or protein levels, have been established based on our understanding of the human genome and various cell signaling pathways. Nucleic acids, peptides/proteins, antibodies, metabolites, lipids, and carbohydrates are among the various biomarkers that have been developed from a variety of specimens, including blood, serum, plasma, body secretions (sputum, saliva), excretions (stool, urine), and circulating tumor cells that are either in the pre-clinical or clinical stages [10].

In recent years, a number of biomarkers for OSCC have been discovered that can be used for diagnosis, differential diagnosis, metastasis prediction, recurrence prediction, and chemotherapy or radiation resistance. For example, Cytokeratin is used as an epithelial tumor marker and has been used as a diagnostic marker for epithelial malignancies including OSCC [12]. More than 90% of OSCC cases have an overexpression of the *EGFR*. This upregulation correlates with cancer growth and progression, resistance to therapy, and poor outcomes in HNSCC patients [12]. In addition, *P53* tumor suppressor gene mutation as well as its reduced expression are predictive biomarkers for recurrence, metastasis, and therapy resistance. Further, increased expression of *Cyclin-D1*, *CD44*, *vascular endothelial growth factor receptor (VEGFR), matrix metalloproteinases (MMPs), integrin α3 and integrin β4, B-cell lymphoma-2(Bcl-2), claudin 4 (CLDN4), yes-associated protein 1 (YAP1), cMET proto-oncogene, chemokine receptors CXCR4, CCR7* and reduced expression of *E-cadherin* are used as OSCC diagnostic/prognostic biomarkers [12,13]. Although, OSCC is less associated with HPV infection, detection of HPV-E6 or E7 oncoproteins and *p16INK4a* markers have been used for the diagnosis of HPV-related oral cancers [12,13]. Because earlier intervention can improve treatment outcomes, these biomarkers can aid in OSCC screening, early identification, and prognosis. To determine cancer pathology and stage, these biomarkers must be utilized in conjunction with other diagnostic techniques as they are inadequate as a stand-alone diagnostic tool. Most of the time, these biomarkers are not type-specific. There are drawbacks and limited effectiveness when using these markers as treatment targets. Different non-coding RNAs have recently demonstrated potential uses in pre-clinical and clinical systems as therapeutic and diagnostic markers. The results of these investigations may indicate that non-coding RNAs are useful as additional biomarkers for oral cancer diagnosis and treatment.

### Long non-coding RNAs in oral cancer

Recent studies reported the importance of lncRNAs in oral cancers. The lncRNAs play important roles in OSCC progression or suppression. By analyzing TCGA head and neck cancer data, one study identified 861 upregulated exonic lncRNAs and 155 downregulated exonic lncRNAs in cancer samples as compared to the normal tissue samples [14]. Following validation in eighteen distinct OSCC cell lines, six lncRNAs—DLEU1, TCONS_00015845, LINC00941, LINC00460, TCONS_00025137, and TCONS_00005474—were found to be highly elevated. Furthermore, through control of CD44 signaling pathways, the DLEU1 demonstrated possible roles in oral cancer proliferation, migration, invasion, and xenograft tumor formation [14]. Data from 167 OSCC samples and 45 normal oral tissues were evaluated in a different study using the Gene Expression Omnibus (GEO) [GSE30784] dataset [15]. This study identified 29 differentially expressed lncRNAs among which 11 genes were upregulated and 18 were downregulated. Further analysis revealed a significant upregulation of lncRNA U62317.1 along with downregulation of LINC01697, LINC02487, LOC105376575, AC005083.1, and SLC8A1-AS1 which were predicted as diagnostic biomarkers [15]. Functional GO and KEGG enrichment analysis showed their involvement in several lipid metabolic processes, such as membrane lipid metabolic process, cellular lipid catabolic process, sphingolipid metabolic process, and fatty acid derivative metabolic process [15]. The function and clinical importance of different lncRNAs in OSCC are summarized in Table 1. As indicated in Table 1, lncRNAs ANRIL, CAS9, CYTOR, DANCR, ELDR, H19, HOTAIR, HOTTIP, IGF2BP2-AS1, LINC00284, LINC00662, LINC0116, LNCAROD, MALAT-1, NEAT1, NORAD, PCAT1, PVT1, ROR, TTN-AS1, TUG1, XIST, UCA1, etc., are upregulated in the OSCC and they regulate cell proliferation, colony formation, invasion, migration, metastasis and drug resistance. On the other hand, lncRNAs FALEC, FENDRR, GAS5, MEG3, MORT, PTCS3, and SCIRT are found to be downregulated and are associated with OSCC suppression. The oncogenic or tumor suppressor lncRNAs interact with miRNAs or RNA binding proteins to regulate different cellular pathways resulting in modulation in OSCC growth and metastasis. A few of these lncRNAs such as lncRNA CAS9, DANCR, HOTAIR, HOTTIP, LINC00284, LINC00668, MALAT-1, NCK1-AS, PVT1, PLAC2, SNHG20, SCIRT, etc., have been examined in human tissues or body fluids and shown potential role as disease biomarkers. However, comprehensive mechanistic validation and clinical significance evaluation in larger patient cohorts are required in this area.

**Table 1:** List of lncRNAs and their significance in OSCC

Name	Study model	Targets/related pathways	Function	Biomarker/therapy	References
**Up-regulated**					
**AC007271.3 ↑**	Tissues, cell lines and nude mice	*Wnt/β-catenin signaling pathway, miR-125b-2-3p/Slug/E-cadherin axis*	Promotes proliferation, invasion and inhibits apoptosis and promotes *in-vivo* tumor growth	Prognosis and therapeutic target	[16,17]
**ADAMTS9-AS2 ↑**	Tissue samples, cell lines and xenograft mouse model	*Binds with miR-143-3p and activates PI3K/Akt and MEK/Erk signaling*	Promotes migration and invasion and facilitated metastasis in salivary adenoid cystic carcinoma (SACC)	Therapeutic target	[18]
**AFAP1-AS1 ↑**	Cell lines, xenograft model	*CCNA2*	Encourages tumor proliferation and indicates a poor prognosis	Unfavorable biomarker, therapeutic target	[19]
**ANRIL ↑**	Tissues, serum and cell lines	*miR-125a*	Promotes proliferation, invasion, and migration	Biomarker (Prognostic)	[20]
**BBOX1 ↑**	Tissue and cell lines	*miR-3940-3p/laminin subunit gamma 2 axis*	Encourages proliferation, and migration and suppresses apoptosis	Therapeutic target	[21]
**BLACAT1 ↑**	Cell lines	*miR-142-5p*	Regulates viability, and causes migration and invasion of cells	Therapeutic target	[22]
**CASC9 ↑**	Tissues, cell lines, *in-vivo* mice model	*AKT/mTOR pathway*	Enhances tumor progression via suppression of autophagy-mediated cell apoptosis	Biomarker (diagnosis and prognosis)	[23]
**CACS15 ↑**	Tissue samples	*MEG3*	Promotes proliferation	Diagnostic biomarker	[24]
**CCAT1 ↑**	Tissue samples, cell lines, mice model	*miR-181a/Wnt/β-catenin signaling*	Promotes proliferation, migration and invasion and suppresses apoptosis	Prognostic and therapeutic biomarker	[25]
**CRNDE ↑**	Tissue samples, cell lines, mice model	*--*	Promotes proliferation, migration and invasion by regulating N-cadherin, vimentin, Snail and Wnt signaling	Therapeutic	[26]
**CYTOR/ LINC00152 ↑**	Tissue samples, cell lines, mice model	*interacts with HNRNPC, miR-1252-5p, miR-3148, and miR-193b-3p/ PI3K-AKT pathway*	Induces cell proliferation, metastasis, drug resistance, mitochondrial metabolism and glycolysis	Prognostic and therapeutic biomarker	[27,28]
**DANCR ↑**	Tissue samples, cell lines	*miR-135a-5p/KLF8 axis, DANCR/miR-4707-3p/FOXC2 pathway*	Promotes proliferation, invasion, and migration, and suppresses apoptosis.	Biomarker (Prognostic and diagnostic), therapeutic target	[29]
**DCST1-AS1 ↑**	Cell lines, tumor xenograft model	*NF-κB pathway*	Causes M2 polarization of tumor-associated macrophages which thereby promotes tumor malignancy and metastasis	Prognostic indicator	[30]
**DLEU1 ↑**	Cell lines	*miR-149/CDK6 axis, CD44 signaling*	Promotes proliferation, invasion, and migration	Therapeutic target	[14,31,32]
**DNM3OS ↑**	Tissues and cell lines	*DNM3OS/miR-204-5p/HIP1 axis*	Modulates cell viability and migration	Therapeutic target	[33]
**ELDR ↑**	Tissue samples, cell lines, and mouse model	*ILF3-cyclin E1 signaling*,	Induces cell proliferation, and inhibits miR-7 to regulate EGFR. Regulates Cyclin E1 signalling through ILF3	Therapeutic target	[34]
**ELF3-AS1↑**	Tissue samples and cell lines	*Induces Glut-1 expression*	Induces glucose uptake and cell proliferation	---	[35]
**EGFR-AS1 ↑**	Tissue samples, cell lines	*miR-145, and ROCK1*	Induces cell proliferation, invasion, and migration	Prognostic and therapeutic	[36]
**FAL1 ↑**	Tissues and cell lines	*miRNA-761/CRKL pathway*	Causes proliferation and develops OSCC	Therapeutic target	[37]
**FTH1P3 ↑**	Tissues and cell lines	*PI3K/ Akt/GSK3b/ Wnt/β-catenin*	Induces cancer cell proliferation, migration, and invasion	Biomarker	[38]
**FOXC2-AS1 ↑**	Tissue samples, cell lines	*miR-6868-5p/E2F3 axis*	Improves proliferation, invasion, migration, and EMT and regulates the cell cycle	Therapeutic target	[39]
**FOXD2-AS1 ↑**	Cell lines	*CDK2, CDK4, and P21*	Induces cell cycle, cell proliferation, and colony formation as well negatively associated with mast cell, dendritic cells (DCs), iDCs, and B cells	Prognostic and Therapeutic biomarker	[40,41]
**GACAT1 ↑**	Tissue samples and cancer cell lines	*miR-149*	Promotes tumor growth and migration	Therapeutic target	[42]
**H19 ↑**	Tissue samples, cell lines, and *in-vivo* mice model	*miR‑138/EZH2 axis, miR-675-5p/PFKFB3 axis*	Promotes proliferation, invasion, migration, and glycolysis	Therapeutic target	[43,44]
**HNF1A-AS1 ↑**	Tissue samples and cell lines	*Notch signaling pathway*	Promotes OSCC progression	Therapeutic target	[45]
**HOTAIR ↑**	Tissue samples and cell lines	*EZH2*	promotes tumor cell invasion and metastasis and represses E-cadherin in OSCC	Biomarker and therapeutic target	[46]
**HOTTIP ↑**	Tissue samples, cell lines, and mice model	*HMGA2-Mediated Wnt/β-Catenin Pathway*	Causes lymph node metastasis. Regulation proliferation, migration, and invasion.	Biomarker (diagnosis) and therapeutic target	[47]
**HOXA-AS2 ↑**	Tissue samples, cell lines, and mice model	*miR-567/CDK8*	Causes OSCC cell proliferation and promotes tumor growth *in-vivo*	Biomarker (prognostic) & therapeutic target	[48]
**HOXA10-AS↑**	Cell lines and mice model	*TP63* mRNA	Promotes oral cancer growth, and metastasis.	Therapeutic target	[49]
**HOXA11-AS ↑**	Tissue samples, cell lines, and mice model	*miR-214-3p/PIM1*	Promotes proliferation, and facilitates CDDP-resistance	Therapeutic marker	[50]
**HIFCAR ↑**	Cell lines and mice model	*Hypoxia-inducible factor (HIF-1α)*	Modulates the hypoxia signal pathway and contributes to OSCC progression, sphere-forming ability, metabolic shift and metastatic potential	Prognostic and therapeutic biomarker	[51]
**IFITM4P ↑**	Tissue samples, cell lines, and mice model	*PD-L1*	Induces cell proliferation and enhanced immune escape	Therapeutic target	[52]
**IGF2BP2-AS1 ↑**	Tissue samples and cell lines	*Wnt/β-catenin pathway*	Promotes cell growth, and migration and restricts apoptosis	Therapeutic target	[53]
**JPX ↑**	Cell lines	*miR-944/CDH2 axis*	Promotes proliferation, invasion, and migration	Therapeutic target	[54]
**KCNQ1OT1↑**	Cisplatin-resistant TSCC samples and TSCC cell lines	*KCNQ1OT1/ miR-124-3p/ TRIM14 axis*	Increases cisplatin resistance, regulates proliferation and metastasis of cisplatin-resistant TSCC		[55]
**LEF1-AS1 ↑**	Tissue samples, cell lines	*LATS1*	Increases cell survival, proliferation, and migration. Retards cell apoptosis	Therapeutic target and biomarker	[56]
**LHFPL3-AS1 ↑**	Tissue samples and cell lines	*LHFPL3-AS1/miR-362-5p/CHSY1 Pathway*	Promotes OSCC growth and cisplatin resistance	–	[57]
**LINC00284 ↑**	Tissue samples, cell lines	*miR-211-3p/MAFG axis*	Causes cell proliferation, migration	Biomarker	[58]
**LINC00319 ↑**	Tissue samples and cancer cells	*miR-199a-5p/FZD4 axis*	Induces proliferation, metastasis, EMT, invasion, and angiogenesis	Therapeutic target	[59]
**LINC00460↑**	Tissue samples, cancer cell lines, and mice model	*Interacts with PRDX1 and facilitates PRDX1 entry into the nucleus*	Induces proliferation, epithelial-mesenchymal transition (EMT), and metastasis *in-vitro* and *in-vivo*	Prognostic and therapeutic biomarker	[60,61]
**LINC00662 ↑**	Tissue samples and cell lines	*miR-144-3p/EZH2 axis*	Increased TNM stage and lymph node metastasis of the patients. Promotes cell growth and metastasis	Therapeutic target	[62]
**LINC00668 ↑**	Tissue samples cell lines	*miR-297/VEGFA axis*	Facilitate VEGFA expression, and promote tumor growth	Biomarker (diagnosis) and therapeutic target	[63]
**LINC00887 ↑**	Tongue squamous carcinoma cell lines and mouse model	*HIF1α or DNMT1*	Variant 887L activates Carbonic Anhydrase IX (CA9)’s transcription by recruiting HIF1α, variant 887S suppresses CA9 through DNMT1-mediated DNA methylation, resulting regulation in proliferation under hypoxic condition	Therapeutic	[64]
**LINC00941↑**	Tissue samples, cancer cell lines, and mice model	*Activates CAPRIN2 promoter and interacts with hnRNPK*	Induces cell proliferation, colony formation, migration, invasion, metastasis and activates WNT/β-catenin signaling pathway	Prognostic and therapeutic markers	[65,66]
**LINC00958 ↑**	Tissue samples and cell lines	*miR-627-5p/YBX2 axis*	Promotes proliferation, migration, EMT and retards apoptosis	Therapeutic target & biomarker (prognostic)	[67]
**LINC00963 ↑**	Tissue samples, cancer cell lines, and mice model	*ABCB5*	Promotes cancer Stemness, increases cancer aggressiveness, and reduces chemosensitivity	Therapeutic target	[68]
**LINC00974 ↑**	Tissue samples, cell lines	*miR-122, RhoA*	Promotes invasion and migration	–	[69]
**LINC01116 ↑**	Tissue samples, cell lines	*LINC01116/miR-9/MMP1 axis*	Causes migration and invasion	Therapeutic target	[70]
**LINC01137 ↑**	Cell lines	*miR-22-3p*	Promotes proliferation, invasion, and migration	Therapeutic target	[71]
**LINC01207 ↑**	Tissue samples and cell lines	*miR-1301-3p/LDHA axis*	Promotes proliferation and migration, reduces apoptosis and autophagy of cells	Novel diagnostic and therapeutic target	[72]
**LINC01234 ↑**	Tissue samples, cell lines, and nude mice model	*miR-637/NUPR1 axis; miR-433/PAK4 axis*	Promotes growth, invasiveness and metastasis	Therapeutic target	[73,74]
**LINC01296 ↑**	Tissue samples, cell lines, and xenograft mice model	SRSF1 protein	Promotes proliferation, invasion, and migration	Therapeutic target	[75]
**LINC02195↑**	Tissue samples, cell lines	---	Increases MHC I protein expression and associated with CD8+ and CD4+ T cell infiltration in microenvironment	Prognostic marker	[76]
**LNCAROD↑**	Tissue samples and cell lines, mice model	Interacts with YBX1 and HSPA1A proteins	Promotes proliferation, invasion, migration, drug resistance	Prognostic and therapeutic biomarker	[77,78]
**MALAT-1 ↑**	Tissue samples, cell lines	*Targets miR101/EZH2; inhibits VHL by EZH2/ STAT3/ Akt axis, induces P-glycoprotein, PI3K/ AKT/ mTOR signaling pathway*	Regulates proliferation, differentiation, and drug resistance through modulation in Wnt/β-catenin signaling, NF-κB signaling, PI3K/AKT/m-TOR signaling pathway	Diagnostic, prognostic, and therapeutic biomarker	[79–82]
**MINCR ↑**	Tissue samples and cancer cell lines	*Wnt/β-catenin pathway*	Causes proliferation and migration	Prognostic biomarker and therapeutic target	[83]
**MIR4435-2HG ↑**	Plasma samples and cell lines	*TGF-β1*	Regulates cancer cell behavior. Involved in the promotion of cancer cell proliferation, migration, and invasion.	Therapeutic target	[84]
**NCK1-AS**	Plasma, tissue, cell lines	*---*	Regulates OSCC cell invasion and migration.	Diagnostic biomarker	[85]
**NEAT1 ↑**	Tissue samples, cell lines, and mice model	*miR-365/RGS20, VEGF-A, and Notch signaling*	Promotes proliferation, migration, and invasion	Therapeutic target	[86–89]
**NORAD ↑**	Tissues and cells	*miR-577/TPM4 axis*	Causes cell proliferation, migration, decreasing apoptosis, sponges miR-577 to enhance TPM4	Therapeutic target	[90]
**OIP5-AS1 ↑**	Tissue samples	*–*	Enhances cancer stemness and is associated with poor clinical outcome		[91]
**ORAOV1-B ↑**	OSCC cells	*Binds to Hsp90 and activates the NF-κB-TNFα loop*.	Induces invasion, migration, and metastasis	Therapeutic target	[92]
**PART1 ↑**	Tissue samples, cell lines, and mice model	*EZH2*	Promotes proliferation, and inhibits apoptosis	Diagnosis biomarker and a novel therapeutic target	[93]
**PCAT-1 ↑**	Tissue samples, cell lines, and mice model	*c-Myc-AKT1-p38 MAPK signaling pathways*	Regulates proliferation, and inhibits apoptosis	Therapeutic target	[94]
**PVT1 ↑**	Tissue samples, cell lines, and mice model	*Wnt/β-catenin signaling, miR-150-5p/GLUT-1*	Increases metastasis, proliferation, and invasion, enhanced EMT and cancer cell stemness	Biomarker and therapeutic target	[95–97]
**PRNCR1 ↑**	Cell lines	*miR-326/FSCN1 axis*	Promotes proliferation, invasion, and migration	–	[98]
**PLAC2 ↑**	Tissue samples, cell lines, and mice model	Downstream *Wnt/β-catenin signaling pathway*	Promotes proliferation and invasion in OSCC cells as well as tumor growth and metastasis *in-vivo*	Biomarker (prognosis and therapy)	[99]
**PTTG3P ↑**	OSCC cells	*PTTG3P/miR-142-5p/JAG1 axis*	Promotes proliferation and migration	–	[100]
**-ROR ↑**	Tissue samples	*miR-145*	Regulates cellular differentiation, represses p53	Prognostic biomarker	[101]
**SLC16A1-AS1 ↑**	Tissue samples, cell lines	*SLC16A1-AS1/CCND1 (requires further elucidation)*	Promotes proliferation, accelerates cell cycle, and modulates immune microenvironment	Therapeutic target & diagnostic indicator	[102]
**SNHG1 ↑**	Cancer cell lines	*miR-421/HMGB2 axis*	Leads to the proliferation of cancer cells	Therapeutic target	[103]
**SNHG6 ↑**	Tca1183 cells	*β-catenin and E-cadherin*	Improves cell viability, proliferation, and EMT. Inhibits apoptosis	–	[104]
**SNHG20 ↑**	Tissue samples, cell lines	*miRNA-19b-3p/RAB14 axis*	Promotes proliferation, migration, and invasion	Biomarker (diagnosis), and therapeutic target	[105]
**SNHG26 ↑**	Tissue samples, cell lines, and mice model	*PGK1/Akt/mTOR*	Promotes TSCC growth, metastasis, and cisplatin resistance	Therapeutic target and biomarker (diagnosis)	[106]
**TTN-AS1 ↑**	Tissue samples, cell lines, and mice model	*miR-411-3p/NFAT5 axis*	Promotes cell growth, and migration and restricts apoptosis	Therapeutic target	[107]
**TSPEAR-AS2 ↑**	Tissue samples,	*TSPEAR-AS2/miR-487a-3p/PPM1A axis*	Promotes tumor cell progression and is associated with advanced TNM staging	Biomarker & therapeutic strategy	[108]
**TUG1 ↑**	Tissue samples, cell lines, and mice model	*miR-593-3p/MAPK axis*	Promotes proliferation, invasion, and migration, prevents apoptosis	Therapeutic target & biomarker (diagnosis)	[109]
**UCA1 ↑**	Tissue samples, cell lines, and mice model	*miR-184, miR-124/TGF-β1/JAG1/Notch axis; miR-143-3p/MYO6 axis; Wnt/β-catenin pathway*	Promotes proliferation, invasion, migration, and cisplatin resistance as well as suppressed apoptosis in OSCC cells	Therapeutic target	[110–112]
**XIST ↑**	Cell lines, and mice model	*miR-29b*	Regulates miR-29b expression, which induces cell apoptosis through the p53 pathway and promotes tumor growth in *in-vivo* model	Therapeutic target	[113]
**ZEB1-AS1 ↑**	Tissue samples, cell lines, and mice model	*miR-23a*	Promotes EMT, cell invasion, and migration. Act as a tumor promoter	Therapeutic target	[114]
**Down-regulated**					
**AC012456.4 ↓**	Tissue samples, cell lines	*JAK-STAT and MAPK signaling pathways*	Significantly associated with tumor staging and survival rates for patients	Diagnostic, therapeutic, and prognostic biomarker	[115]
**FALEC↓**	TSCC tissue sample, cell lines, and mouse model	*Recruits EZH2 at the promoter regions of ECM1 repressing ECM1 expression*.	Inhibits cell proliferation and migration	Therapeutic and prognostic biomarker	[116]
**FENDRR ↓**	Tissue samples, cell lines	*PI3K/AKT pathway*	Inhibits angiogenesis of OSCC	Therapeutic target	[117]
**GAS5 ↓**	Cell lines	*miR-21/PTEN axis*	Inhibits proliferation, invasion, EMT, and migration	Therapeutic target	[118]
**HCG11 ↓**	Cell lines	*miR-455-5p*	Reduces OSCC proliferation, increased G1/S transition, and Ki67 levels	Therapeutic target	[119]
**MEG3 ↓**	Tissue samples, cell lines	*miR-421, miR-21, miR-548d-3p/SOCS5-SOCS6/JAK/STAT, Wnt/β-catenin pathway*	Down-regulation alleviates the aggressiveness of cancer and is associated with poor prognosis. Reduces tumor growth by inhibiting cell proliferation, cell cycle, and metastasis, and induces cell apoptosis	Therapeutic and prognostic biomarker	[120–123]
**MORT ↓**	Tissue samples, cell lines	*ROCK1*	Inhibits proliferation and is correlated with poor survival	Therapeutic target	[124]
**PTCSC3 ↓**	Tissue samples, cell lines	*—*	Promotes apoptosis and autophagy and suppresses cancer cell proliferation	Therapeutic target	[125]
**SCIRT ↓**	Tissue samples	*miR-221*	Induces cancer cell apoptosis	Biomarker	[126]

Note: Up arrows indicate up-regulation and down arrows indicate down-regulation.

### Role of long non-coding RNA in OSCC

LncRNAs regulate multiple cellular mechanisms during OSCC progression depending on where they are located. Most lncRNAs are expressed in the nucleus, where they control epigenetic modifications, chromatin conformation, and nuclear shape. Moreover, they interact with DNA to produce R-loops, which control transcription and maintain the integrity of the genome [7,8]. For instance, LncRNA Linc-Pint (long intergenic non-protein coding RNA, p53-induced transcript) (chromosome 7q32.3) directly interacts with the nucleus’s serine/arginine protein kinase 2 (SRPK2) and Polycomb repressive complex 2 (PRC2) to regulate splicing and PRC2-mediated H3K27 trimethylation as well as SRPK2-mediated SR-protein phosphorylation [127,128]. Nuclear lncRNAs that control nuclear structure, splicing, epigenetic modification, and synapse formation are MALAT-1 (metastasis-associated lung adenocarcinoma transcript 1) (chromosome 11q13.1), NEAT1 (nuclear paraspeckle assembly transcript 1) (chromosome 11q13.1), and XIST (X-inactive-specific transcript) (chromosome Xq13.2) [7]. Conversely, lncRNAs interact with protein and miRNAs in the cytoplasm to control mRNA translation and stability [7,8]. For instance, PUMILIO proteins PUM-1 and PUM-2 are sequestered in the cytoplasm by lncRNA NORAD (Noncoding RNA activated by DNA damage), which also inhibits PUM-1/2-driven chromosomal segregation, genomic instability, and mRNA destabilization [129]. Additionally, during hepatitis C virus infection, NORAD interacts with miR-373 to stabilize the miR-373 target mRNA Wee1 in hepatocytes [130]. Conversely, several biological processes are regulated by GAS5 (growth arrest-specific 5) (chromosome 1q25.1), HOTAIR (HOX antisense intergenic RNA) (chromosome 12q13.13), and FIRRE (FIRRE intergenic repeating RNA element) (chromosome Xq26.2), which are found in both the nucleus and the cytoplasm [7].

**Figure 1:**
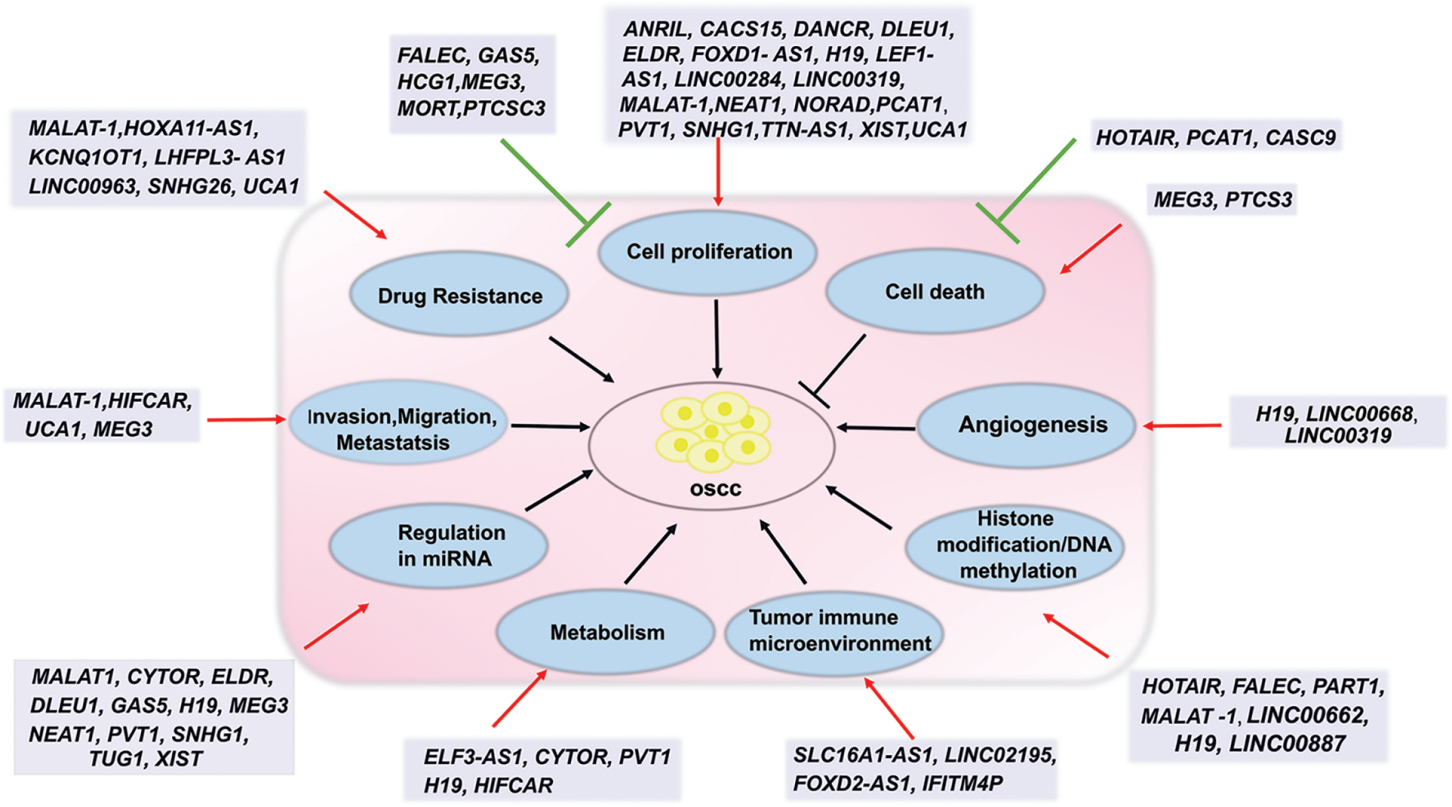
Diagrammatic illustration of the function lncRNAs in the regulation of several signaling pathways and events in OSCC. Sharp arrows indicate induction/activation; blunt arrows indicate inhibition.

In OSCC, dysregulation of lncRNAs leads to the regulation of many cellular pathways. LncRNAs bind to DNA, RNA, proteins, or miRNA and thus regulate transcription, post-transcription, translation, and post-translational events depending on their concentration and localization in cells. Upregulation of oncogenic lncRNAs and down-regulation of tumor-suppressor lncRNAs favor multiple cellular events that are associated with OSCC cell survival, proliferation, invasion, migration, metastasis, angiogenesis, metabolism, epigenetic modification, tumor immune microenvironment and drug resistance. A single lncRNA has multiple targets. For example, MALAT-1 is one of the well-studied lncRNA and is frequently upregulated in OSCC. Analysis of TCGA head and neck cancer data shows that the MALAT-1 gene is upregulated in all the stages (http://gepia.cancer-pku.cn/). In-silico analysis shows that MALAT-1 interacts with around 7856 protein-coding genes and more than 350 miRNAs (ENCORI/starBase: rnasysu.com/encori/index.php). Further pathway analysis using PANTHER18.0 (https://pantherdb.org/) shows these protein-coding genes are associated with different signaling pathways including Wnt signaling pathway (P00057), Integrin signaling pathway (P00034), Angiogenesis (P00005), EGF receptor signaling pathway (P00018), Apoptosis signaling pathway (P00006), p53 pathway (P00059), T cell activation (P00053), Ras Pathway (P04393), B cell activation (P00010), etc. In addition, the MALAT-1 is involved in lncRNA-mediated post-transcriptional gene silencing, cellular response to hypoxia, positive regulation of cardiac muscle myoblast proliferation, positive regulation of cell motility, and positive regulation of miRNA catabolic process (https://www.genecards.org/). Further studies show that MALAT-1 targets the miR101/EZH2 axis, inhibits VHL by EZH2/STAT3/Akt axis, induces P-glycoprotein, Wnt/β-catenin signaling, NF-κB signaling, and PI3K/AKT/mTOR signaling pathways in OSCC resulting in regulation of OSCC survival, proliferation, differentiation, metastasis and drug resistance (Table 1). NEAT1 is elevated over the whole OSCC spectrum (http://gepia.cancer-pku.cn/). According to ENCORI/starBase (rnasysu.com/encori/index.php), NEAT1 interacts with approximately 6403 protein-coding genes and 1236 miRNAs. The majority of the genes that code for proteins are linked to several pathways, such as the “Integrin signalling pathway (P00034),” “Wnt signaling pathway (P00057),” and the “Gonadotropin-releasing hormone receptor pathway (P06664)” (PANTHER18.0: https://pantherdb.org/). Further, mechanistic studies show that NEAT1 induces OSCC proliferation, migration, and invasion by targeting the miR-365/RGS20 axis, VEGF-A signaling, and Notch signaling (Table 1). Another lncRNA ELDR is upregulated in OSCC and this upregulation is associated with cancer stages [34]. RNA pull-down analysis followed by mass spectrometry using ELDR sense *vs*. anti-sense strand identified 4495 interacting proteins [34]. Using GENEONTOLOGY PANTHER analysis, 444 proteins were categorized as having “Molecular Function,” with 240 of those proteins having an RNA binding function (GO: 0005488). Further mechanistic investigation showed that the ELDR regulates OSCC proliferation by inducing EGFR signaling and Cyclin E1 signaling (Table 1). On the other hand, the lncRNAs GAS5 and MEG3 are downregulated in OSCC. Mechanistically GAS5 inhibits OSCC proliferation, invasion, EMT, and migration (Table 1). The down regulation of MEG3 is associated with OSCC aggressiveness and poor prognosis. It reduces tumor proliferation and metastasis and induces apoptosis (Table 1). Thus, lncRNAs interplay multiple cellular pathways resulting in OSCC progression, survival, metastasis, and drug resistance. Their functions in OSCC are outlined in detail below and summarized in Fig. 1.

### LncRNAs in OSCC cell proliferation

Deregulation of lncRNAs in OSCC is associated with the modulation in cell proliferation, cell cycle, and cell signaling. In OSCC, lncRNAs are found to regulate different cell proliferation pathways including EGFR, PI3K/AKT/mTOR, STAT3, JAG, NF-kB, and Wnt signaling. For example, MALAT-1 is upregulated in OSCC. Depletion of MALAT-1 by siRNA resulted in inhibition of cell growth *in-vitro* and *in-vivo*, and G1 phase cell cycle arrest [79]. Mechanistically, MALAT-1 suppressed von Hippel-Lindau tumor suppressor protein (VHL) by activating the EZH2/STAT3/Akt axis resulting in the stabilization of β-catenin and NF-κB, both of which play important roles in OSCC progression. The MALAT-1 also upregulates PI3K/AKT/mTOR, MMP9, and Wnt/β-catenin signaling pathways [80]. On the other hand, MALAT-1 interacts with multiple miRNAs resulting prevention of miRNA and its target mRNA interaction. For example, MALAT-1 regulates miR-125b/STAT3, miRNA-320d/SRSF1, and miR-124-/JAG signaling in OSCC [80]. Another lncRNA ELDR is upregulated in OSCC and depletion of ELDR inhibits *in-vitro* and *in-vivo* tumor growth and induces G1/S cell cycle arrest [34]. The ELDR regulates OSCC cell proliferation by interacting with RNA binding protein ILF3 resulting in the stabilization of *Cyclin E1* [34]. Further, the ELDR inhibits miR-7 and thus stabilizes *EGFR* in the regulation of cell proliferation [34]. In normal oral keratinocytes (NOKs), ELDR over-expression induces cell proliferation and G2/M phase cell cycle progression by inducing CTCF/FOXM1/AURKA signaling [131]. This suggests that the ELDR may be one of the driver genes of oral carcinogenesis. LncRNA PVT1 is upregulated in OSCC and induces cell proliferation by interacting with miR-150-5p and inducing Wnt signaling [95–97]. As stated in Table 1 and Fig. 1, several upregulated lncRNAs including ANRIL, CACS15, DANCR, DLEU1, FOXD1-AS1, H19, LEF1-AS1, LINC00284, LINC00319, NEAT1, NORAD, PCAT1, SNHG1, TTN-AS1, XIST, and UCA1 induce OSCC cell proliferation. On the other hand, the lncRNA MEG3 is down-regulated by H3K27me3 modification in OSCC [132]. Over-expression of MEG3 inhibited OSCC proliferation by reducing the Wnt signaling pathway via *GATA3* and interacting with miR-21 [121,132]. Another lncRNA MORT is downregulated in OSCC [124]. Over-expression of MORT inhibits OSCC cell proliferation by downregulating *ROCK1* [124]. Thus, several lncRNAs play important roles in OSCC growth.

### LncRNAs in OSCC cell death

Dysregulation of the apoptotic pathway by genetic or epigenetic mechanisms appears to be one of the key mechanisms by which cancer cells avoid cell death [133]. Upregulation of PI3K-AKT-mTOR axis, BCl2 proteins, and down-regulation of Fas receptor, BAX, and Caspases 3, 8, 9 lead to anti-apoptotic signaling in OSCC [133]. On the other hand, the mTOR kinase and the PI3K/AKT survival pathway both influence autophagy [134]. Because of its ability to either trigger cell death or enhance cell survival, significant evidence suggests that autophagy plays a dual role in OSCC [134]. LncRNAs regulate either apoptosis or autophagy or both to control cell death. As stated in Table 1 and Fig. 1, many oncogenic lncRNAs provide OSCC with a pro-survival signal, whereas tumor suppressor lncRNAs have the potential to induce apoptosis in OSCC. LncRNAs regulate cell survival in multiple ways. For example, lncRNA HOTAIR is upregulated in OSCC. Silencing of HOTAIR by siRNA inhibits autophagy by reducing expression of *MAP1LC3B* (microtubule-associated protein 1 light chain 3B), beclin1, and autophagy-related gene 3 (*ATG3*) and *ATG7* [135]. On the other hand, HOTAIR depletion promotes apoptosis through mTOR mediated pathway [135]. Mechanistically HOTAIR interacts with EZH2 which suppresses pro-apoptotic gene expression like *BIM1* by histone methylation [46,136]. The PCAT-1 knockdown activates p21, p38/MAPK, and apoptosis signal-regulating kinase 1, resulting in Caspase 9 and PARP-mediated apoptosis [94,137]. Mechanistically, PCAT-1 interacts with EZH2 and thus represses *p21* expression [138]. The CASC9 is upregulated in OSCC. The knockdown of CASC9 induces both autophagy and apoptosis while decreasing proliferation in OSCC cells SCC15 and CAL27. Furthermore, p-AKT, p-mTOR, P62, and BCL-2 expression levels were considerably reduced, although BAX expression levels and the LC3BI/LC3BII ratio were elevated in CASC9-knockdown OSCC cells [23]. The PTCSC3 is down-regulated in OSCC [125]. PTCSC3 over-expression reduced the growth of human OSCC cells by inducing autophagy and apoptosis through the increase in LC3B-I, Beclin1, and Bax expression and inhibition of Bcl-2 [125]. The lncRNA MEG3 inhibits miR-21 and thus induces expression of *PTEN*, resulting in the induction of apoptosis [121,139].

In contrast to apoptosis, and autophagy, ferroptosis is another type of regulated cell death that is dependent on iron metabolism, generation of reactive oxygen species [ROS], and lipid peroxidation [140,141]. Ferroptosis has been shown to have a significant impact on OSCC [140]. Inducing ferroptosis in cancer cells has emerged as a potential target for cancer therapy, especially in cases of therapy-resistant cancers [140]. Recent studies show the importance of non-coding RNAs in the regulation of ferroptosis in OSCC or HNSCC, but little is known about the underlying molecular pathways [141]. In a study, 935 ferroptosis-related lncRNAs were discovered using correlation analysis between TCGA head and neck cancer data (42 normal and 487 tumors) and ferroptosis markers from the FerrDb database [142]. Among these, 25 lncRNAs were differentially expressed including AATBC, AC007991.2, AC008115.3, AC012467.2, AC012640.2, AC104083.1, AC106820.3, AC116914.2, AC135050.6, AC136475.2, AC144831.1, AL022328.2, AL132989.1, AL139158.2, AL161431.1, AL357033.4, AL450992.2, AL451085.2, ELF3-AS1, EP300-AS1, LINC01963, LINC01980, PAX8-AS1, PCED1B-AS1, and PSMA3-AS1. These lncRNAs are linked to the prognosis of cancer and primarily control immune system functions, indicating that ferroptosis and immune checkpoint inhibitors may work in combination to increase the antitumor effect [142]. Another study identified 363 lncRNAs associated with both autophagy and ferroptosis using the TCGA head and neck cancer data [143]. Among these, 17 lncRNAs were found to be associated with the survival of the patients, and the lncRNAs PCED1B-AS1, AL512274.1, AL354836.1, MIR9-3HG, MIR4435-2HG, and LINC02541 were found to be independent factors in head and neck cancer [143]. In OSCC patient samples from the TCGA database, 377 differentially expressed lncRNAs associated with ferroptosis were found in another study [144]. LncRNAs STARD4-AS1, AC099850.3, AC090246.1, ALMS1-IT1, AC021087.4, MIAT, HOTAIRM1, and AL512274.1 were among those that demonstrated prognostic significance in OSCC. Ferroptosis-associated lncRNAs were identified in a related study as FIRRE, LINC01305, AC099850.3, AL512274.1, AC090246.1, MIAT, AC079921.2, and LINC00524 [145]. However, to fully understand the molecular mechanism of lncRNAs in ferroptosis, more mechanistic research is necessary. Thus, lncRNAs play an important role in OSCC cell survival and development.

### LncRNAs in cell invasion, migration, and metastasis

Most tumor cells show persistent invasion, which often results in distant lymphatic metastasis and local recurrence [146]. Invasion includes the simultaneous infiltration and destruction of neighboring tissues. The process is associated with potential changes in cell adhesion receptor expression, and their related signaling pathways including Epithelial-Mesenchymal transition (EMT), TGFβ-1, EGF/EGFR, Wnt/β-Catenin, NF-κB, STAT3 and PI3K/Akt which lead to a highly invasive and metastatic character [112,117,146,147]. Modification of the tumor microenvironment (TME) with changes in the extracellular matrix (ECM) plays a crucial role in cell migration and metastasis [147]. A number of lncRNAs directly or indirectly regulate OSCC invasion, migration, and metastasis [112] (Fig. 1). Several studies have demonstrated that lncRNAs can influence tumor metastasis by controlling a range of biological processes, including chromatin modification, transcriptional or translational regulation, mRNA stabilization, inducing EMT, and interaction with miRNAs [112]. Nuclear MALAT-1 could reinforce trimethylation of histone H3 lysine27 (H3K27me3) to decrease gene transcription in an EZH2-dependent way [79]. As discussed above, the MALAT-1-mediated suppression of VHL by activating the EZH2/STAT3/Akt axis induces growth and metastasis [79]. The *EZH2* gene has been identified as a direct target of miR-101 in a variety of malignancies [81]. MALAT-1 promotes OSCC proliferation and invasion by targeting the miR101/EZH2 axis [81]. Another lncRNA H19 induces *EZH2* by inhibiting its target miR-138 in OSCC and thus promotes cell proliferation and invasion [43]. Extensive clinical data analysis shows that HIFCAR (MIR31HG) is significantly upregulated in OSCC [51]. Mechanistically, the HIFCAR forms a complex with transcription factor HIF-1α through direct binding and enhances the recruitment of HIF-1α and the p300 cofactor to target promoters resulting in OSCC proliferation and metastasis [51]. In tongue cancer tissues and cell lines, UCA1 is shown to be over-expressed [111]. The UCA1 knock-down significantly reduced TGFβ-1-induced tongue cancer cell invasion and EMT by reducing vimentin and inducing E-cadherin. The UCA1 could directly bind to miR-124 resulting in induction of miR-124 downstream jagged 1 (*JAG1*) and Notch signaling [111]. In addition, UCA1 targets the miR-143-3p/MYO6 axis and induces the Wnt/β-catenin pathway in the regulation of cancer cell invasion and migration [112]. The lncRNA MEG3 targets miR-421 by sponging and thus induces *E-cadherin* expression in the prevention of OSCC cell proliferation, invasion, migration, and EMT [122]. In addition, MEG3 targets miR-548d-3p/SOCS5-SOCS6/JAK/STAT axis, miR-21, and the Wnt/β-Catenin pathway to inhibit cell proliferation and migration [112]. Thus, many lncRNAs play important roles in OSCC invasion, migration, and metastasis and are predicted to be prognostic or therapeutic biomarkers (Table 1, Fig. 1). However, further mechanistic studies are necessary to understand their roles in the interaction between OSCC cells and the TME.

### LncRNAs in angiogenesis

Angiogenesis is a critical process in the development, recurrence, and metastasis of solid tumors. A vast amount of research indicates that inhibiting angiogenesis could be beneficial in the treatment of solid tumors [148]. However, despite promising preclinical outcomes, anti-angiogenic medicines have shown significant toxicity and limited therapeutic success in head and neck cancer patients [148]. Several molecules including CD44, HIF-1α, HIF-2α, plasminogen activator inhibitor-1 (PAI-1), CXC chemokine receptor 7 (CXCR7), MMPs (MMP7, 9), NOTCH signaling, VEGF and its receptors (VEGFR-1, -2 and -3), Fibroblast Growth Factor Receptors (FGFRs) play important roles in angiogenesis and deregulated expression of such molecules are evident in HNSCC and OSCC [149]. Deregulated expression of lncRNAs is found to be associated with angiogenesis and is suggested to play an important role in diagnosis and therapy [150]. For example, lncRNAs LINC00173.v1, MYLK-AS1, MALAT-1, SNHG17, SNHG22, TUG1, SRRM2-AS, LINC01314, EPIC1, RAB11B-AS1, TUG1, LINC00261, SLC26A4-AS1, LINC00320, H19 are found to be associated with angiogenesis in different cancer types [150]. However, the role of lncRNAs in angiogenesis is not well examined in OSCC. The lncRNA H19 suppresses miR-29, miR-30a, miR-107, miR-140, miR-148b, miR-199a and miR-200, which modulate angiogenesis by inhibiting angiogenesis pathway genes *BiP, DLL4, FGF7, HIF1A, HIF1B, HIF2A, PDGFB, PDGFRA, VEGFA, VEGFB, VEGFC, VEGFR1, VEGFR2* and *VEGFR3* in smoking induced systems [151]. The LINC00668 is upregulated in OSCC and acts as a ceRNA for miR-297 and thus induces miR-297 target gene *VEGFA* expression in OSCC development [151]. The LINC00319 is upregulated in oral cancer. Depletion of LINC00319 reduces proliferation, metastasis, EMT, and angiogenesis [59]. Mechanistically, LINC00319 acts as a ceRNA of miR-199a-5p and thus induces the expression of *FZD4* [59]. In a different study, the importance of *FZD4* was described in angiogenesis [152]. In summary, the lncRNAs may have crucial role in the angiogenesis of OSCC (Table 1, Fig. 1). However, further mechanistic studies are needed to understand their role in this regard.

### LncRNAs in metabolism

Tumor cells undergo metabolic reprogramming in order to adapt to the multiple challenges they encounter, which include hypoxia, energy restriction, and an acidic environment. Tumors with varying tissue origins, genetic backgrounds, and clinical stages have diverse metabolic characteristics [153]. One of the key characteristics of OSCC is metabolic reprogramming, which involves changes in the glucose metabolism and Warburg effect, amino acid, and lipid metabolism. Studies show the importance of such metabolites and their regulatory genes in identifying the biomarkers of the disease for an early and accurate diagnosis as well as therapy. LncRNAs are found to play important roles in regulation of cancer metabolism. For example, lncRNAs MACC1‑AS1 and FTX regulate glucose transporter *GLUT-1* in gastric cancer and hepatocellular carcinoma (HCC), respectively; PVT1 and TUG1 regulate glycolysis enzyme *Hexokinase 2 (HK2)* in Osteosarcoma and HCC respectively; LINC-p21, FEZF1‑AS1 and UCA1 regulate another glycolysis enzyme *Pyruvate kinase 2 (PKM2)* in prostate, colorectal and liver cancers respectively; H19 and CASC9 regulates *HIF1α* in breast and Nasopharyngeal carcinoma, respectively; MALAT-1 regulates glucose metabolism gene *TCF7L2* (transcription factor-7-like 2) and NEAT1 regulates lipid metabolism gene *ATGL* (Adipose triglyceride lipase) in HCC [154]. As summarized in Table 1 and Fig. 1, many lncRNAs are found to regulate OSCC metabolism. The lncRNA ELF3-AS1 is overexpressed in OSCC and enhances glucose transporter *GLUT-1* expression, leading to increased glucose uptake and cell proliferation [35]. Another lncRNA CYTOR is upregulated in OSCC. The CYTOR positively regulates mitochondrial functions, and glycolysis in OSCC cells [28]. Mechanistically, CYTOR physically interacts with and stabilizes HNRNPC resulting in HNRNPC-mediated ZEB1 mRNA stabilization which transcriptionally activates *SIRT3* and *COX10*, regulator genes of mitochondrial function and glycolysis [28]. Targeting CYTOR by siRNA significantly inhibits OSCC growth in mouse models [28]. The lncRNA PVT-1 is upregulated in OSCC. The PVT1 sponges miR-150-5p, resulting in the induction of miR-150-5p target gene *GLUT-1* in OSCC [96]. The lncRNA H19 is upregulated in oral cancer-associated fibroblasts (CAFs) and induces glycolysis by increasing the expression of *fructose-2,6-biphosphatase 3 (PFKFB3)* [44]. The CAFs are the dominant population in the tumor microenvironment and regulate tumor development and therapy resistance. The HIFCAR (MIR31HG) is markedly elevated in OSCC and is correlated with unfavorable clinical outcomes. In terms of mechanism, The HIFCAR directly binds to HIF-1α to form a complex, which then helps to recruit HIF-1α and the p300 cofactor to the target promoters resulting in induction of OSCC sphere-forming ability, metabolic shift, and metastatic potential [51]. Genes implicated in angiogenesis, cell survival, glucose metabolism, invasion, and other critical facets of cancer biology are activated transcriptionally by HIF-1α [51,155]. LncRNAs are therefore crucial to OSCC metabolism.

### LncRNAs in epigenetic modifications

Genetic changes alone cannot fully explain the complexity of carcinogenesis; epigenetic modifications, such as alterations to DNA methylation, histone modifications, and miRNAs also play important roles. Aberrant epigenetic alterations play a crucial role in the advancement of cancer, and they most likely arise very early in the genesis of neoplasms [156]. LncRNAs are found to regulate the human genome epigenetically in normal and diseased conditions [157]. For example, DNMT1 associated lncRNA DACOR1 induces DNA methylation at multiple loci without affecting DNMT1 protein levels; HOTAIR and LINC-PINT interact with Polycomb Repressive Complex 2 (PRC2) to silence genes in different cancers [157]. The EZH2, a PRC2 catalytic component and histone methyltransferase, catalyzes the tri-methylation of histone H3 at Lys 27 (H3K27me3) to control the expression of genes by the action of epigenetic machinery [158]. Depending on H3K27me3 levels and various physiological settings, EZH2 can operate as a transcriptional co-activator or suppressor [158]. The EZH2 inhibitors have been the subject of much pre-clinical and clinical research because EZH2 is a possible target for cancer therapy [158]. As summarized in Table 1 and Fig. 1, certain lncRNAs have been shown to control EZH2 in OSCC, either by physically bringing it to the target gene promoter or by stabilizing it, which in turn controls the expression of target genes. The HOTAIR recruits EZH2 in the *E-cadherin* promoter and silences the *E-cadherin* gene through histone methylation (H3K27me3) in OSCC [46]. Similarly, lncRNA FALEC inhibits tongue squamous cell carcinoma (TSCC) proliferation and metastasis by epigenetically repressing extracellular matrix protein 1 (ECM1) through the recruitment of EZH2 at the promoter regions [116]. Through the inhibition of EZH2 degradation, lncRNA PART1 stimulates proliferation and prevents apoptosis in OSCC [93]. The MALAT-1 represses VHL by activating *EZH2*, hence promoting the growth and metastasis of head and neck squamous cell carcinoma [79]. LINC00662 is an oncogenic lncRNA of OSCC and induces expression of *EZH2* by inhibiting its regulator miR-144-3p [62]. The H19 functions as a ceRNA of miR-138 and releases *EZH2*, which stimulates cell proliferation and invasion of OSCC [43]. On the other hand, the LINC00887 variant 887S suppresses Carbonic Anhydrase IX (CA9) transcription upon hypoxia in TSCC progression through DNMT1-mediated DNA methylation [64].

As epigenetic modulators, miRNAs alter the target mRNAs’ without changing the gene sequences [159]. The lncRNAs act as miRNA sponges, compete with miRNA for the common target genes, or downregulate miRNA expression and thus regulate gene expression epigenetically in different cancers [160]. A single lncRNA can interact with multiple miRNAs and multiple lncRNAs may target a single miRNA. For example: The MALAT-1 interacts with more than 350 miRNAs including miR-17-5p, miR-20a-5p, miR-30e-5p, miR-101-3p, miR-150-5p etc.; on the other hand, miR-21-5p interacts with lncRNAs LINC00472, RNF216P1, SNHG1, SNHG14, XIST in different cancers (ENCORI/starBase: rnasysu.com/encori/index.php). Furthermore, a single miRNA targets multiple mRNAs at a time. For example: miR-7-5p has around 5461 target mRNAs in different cancers (ENCORI/starBase). The ELDR inhibits miR-7 in OSCC resulting stabilization of miR-7 target gene *EGFR* in induction of OSCC cell proliferation [34]. The MALAT-1 induces OSCC proliferation and invasion by targeting miR-101 which results in the stabilization of miR-101 target gene EZH2 [81]. CYTOR targets miR-1252-5p, miR-3148, and miR-193b-3p; DLEU1 targets miR-149; GAS5 targets miR-21; H19 targets miR-138; MEG3 targets miR-21, miR-421, and miR-548d-3p; NEAT1 targets miR-365; PVT1 targets miR-150-5p; SNHG1 targets miR-421; TUG1 targets miR-593-3p; XIST targets miR-29b in OSCC (Table 1). The interactions of lncRNAs with miRNAs are summarized in Table 1 and Fig. 1. Thus, the lncRNA/miRNA axis plays a crucial role in OSCC development and may be an important target for OSCC diagnosis and therapy.

### LncRNAs in regulation of immune system

The main constituents of the immune microenvironment are myeloid cells, which include neutrophils, macrophages, and myeloid inhibitory cells (MDSCs), and lymphocytes, which include B cells, natural killer (NK) cells, CD4+ and CD8+ T cells, and regulatory T cells (Tregs) [161]. The level and composition of Immune cells vary between patients with the same tumor type as well as between tumor types. Based on immune cell infiltration, both suppressive and activating immunological phenotypes have been identified in the tumor microenvironment (TME) [161]. LncRNAs have been shown to support or inhibit OSCC growth by taking part in a number of immune response pathways within the TME [162] (Table 1, Fig. 1). But little is known about the mechanism. Bioinformatics analysis using ESTIMATE and CIBSORTE algorithms showed that the lncRNA SLC16A1-AS1 is negatively correlated with the TME, which included immune cells and stromal cells in OSCC [102]. Further, Plasma cells, T follicular cells, resting mast cells, and Treg cells showed negative correlations with the SLC16A1-AS1, while resting NK cells, M1 macrophages, activated mast cells, and activated memory CD4+ T cells showed positive correlations [102]. The LINC02195 is over-expressed in head and neck cancer, and it acts as a favorable prognostic marker [76]. Further analysis revealed that the lncRNA induces the immune system by positively regulating MHC I protein expression and CD8+ and CD4+ T cells within the TME [76]. In OSCC, FOXD2-AS1 is negatively correlated with mast cells, dendritic cells (DCs), iDCs, and B cells [41]. It also plays a role in tumor growth through EMT and cell cycle regulation [41]. The lncRNA IFITM4P is over-expressed in both the cytoplasm and nucleus of OSCC and induces programmed death-ligand 1 (PD-L1) expression resulting in activation of immune suppression [52]. Mechanistically, the IFITM4P recruits SAM and SH3-domain containing protein 1 (SASH1) to bind and phosphorylate TAK1 (MAP3K7) (Thr187) which in turn, increases PD-L1 expression through increased phosphorylation of NF-κB (Ser536). On the other hand, IFITM4P increases KDM5A’s binding to the *PTEN* promoter in the nucleus, resulting in decreased expression of *PTEN* and induction of PD-L1 [52]. Furthermore, animals treated with a programmed cell death protein 1 (PD-1) monoclonal antibody (mAb) demonstrated substantial therapeutic responsiveness when their tumors exhibited high expression levels of IFITM4P [52].

### LncRNAs in drug resistance

Traditional medicines may not always be effective in treating tumors, and as drug-resistant cancers become more common, more study and therapy development are required. Even though many cancer types are initially responsive to chemotherapy, over time, they may become resistant due to activation of cancer stem cells, changes in metabolism, genetic or epigenetic modifications, increased expression of certain ATP-binding cassette (ABC) transport proteins and drug efflux, drug inactivation, drug target alteration, alteration in DNA damage repair, inhibition of cell death, EMT, and intra-tumor heterogeneity [163,164]. LncRNAs positively or negatively regulate one of such mechanisms and interfere with cancer cell drug resistance [164]. For example: PVT-1 is highly expressed in cisplatin resistant gastric cancer patient samples, and its overexpression contributes to the development of multidrug resistance (MDR) in gastric cancer cells [165]. The H19 is upregulated in doxorubicin-resistant HCC [166]. Silencing of H19 inhibits the MDR1/P-glycoprotein expression by increasing promoter methylation, which increases the cellular doxorubicin accumulation level and sensitizes doxorubicin toxicity in both wild-type and resistant cells [166].

In OSCC, the MALAT-1 is upregulated in cisplatin-resistant OSCC cells [82]. Silencing of MALAT-1 induces apoptotic cell death and cisplatin sensitivity by inhibiting P-glycoprotein expression through the PI3K/AKT/mTOR signaling pathway [82]. HOXA11-AS is upregulated in cisplatin-resistant OSCC cells [50]. The upregulation of HOXA11-AS decreases cisplatin-induced cytotoxicity and enhances cell growth in cisplatin-sensitive cells. Conversely, downregulation of HOXA11-AS facilitates CDDP-induced cytotoxicity and reduces proliferation in CDDP-resistant cells. Mechanistically, the HOXA11-AS induces proto-oncogene serine/threonine-kinase (*PIM1*) through targeting miR-214-3p resulting in increased proliferation and drug resistance [50]. Similarly, in cisplatin-resistant tongue cancer cells, the lncRNA KCNQ1OT1 is increased and causes the resistant cells to proliferate, migrate, invade, and undergo EMT [55]. Mechanistically, KCNQ1OT1 promotes *TRIM14* expression by suppressing the *TRIM14* target miR-124-3p [55]. Another lncRNA LHFPL3-AS1 facilitates OSCC proliferation, migration, invasion, and cisplatin resistance by acting as competing endogenous RNA (ceRNA) for miR-194-5p to enhance *Chondroitin Sulfate Synthase 1 (CHSY1)* expression [57]. LINC00963 enhances OSCC stemness, migration, invasion, colony formation capability, and drug resistance by upregulating the expression of the multidrug-resistant transporter *ABCB5* [68]. This suggests that in patients with oral cancer, suppressing LINC00963 may be useful in preventing chemoresistance and cancer relapse. The expression of SNHG26 shows a favorable correlation with the TSCC proliferation invasion, migration, EMT, and cisplatin resistance [106]. The SNHG26 directly binds to the phosphoglycerate kinase 1 (PGK1) protein in TSCC cells, preventing its ubiquitination and triggering the Akt/mTOR signaling cascade [106]. The UCA1 is upregulated in cisplatin-resistant OSCC cells and promotes proliferation and inhibits apoptosis via regulating *SF1* through sponging miR-184 [110]. Suppression of UCA1 regressed OSCC growth in mice models and enhanced cisplatin sensitivity [110]. The role of lncRNAs in drug resistance is summarized in Table 1 and Fig. 1.

### Therapeutic efficacy of long non-coding RNA in oral cancer

The therapeutic efficacy of lncRNAs is studied in mouse xenograft models. Knock-down of oncogenic lncRNAs by shRNA or siRNA and over-expression of tumor suppressor lncRNAs exhibit tumor regression effect in mice models. For example, the lncRNA ADAMTS9-AS2 is highly expressed in salivary adenoid cystic carcinoma (SACC) patients’ samples compared with the adjacent tissues [18]. The lncRNA is associated with prognosis and distant metastasis and induces cell migration and invasion by interacting with miR-143-3p. The therapeutic significance of lncRNA was demonstrated by the reduction in *in-vivo* tumor development and lung metastasis in nude mice when the ADAMTS9-AS2 gene was knocked down in SACC cells using shRNA [18]. The lncRNA CCAT1 is upregulated in OSCC tissues and cell lines [25]. In nude mice, CCAT1 knock-down with shRNA could prevent OSCC tumor growth by blocking Wnt signaling through interaction with miR-181a [25]. The CRNDE expression is upregulated in OSCC tissues and cell lines [26]. It regulates cell proliferation, migration, and invasion by inducing N-cadherin, vimentin, and Snail, and inhibiting. Compared to tumor-bearing nude mice in the control group, the sh-CRNDE group exhibited slowed tumor growth, reduced tumor weight, and increased E-cadherin expression. Additionally, the sh-CRNDE group showed decreased expression of N-cadherin, vimentin, and Snail, indicating a reversal of the epithelial-to-mesenchymal transition (EMT) process [26]. The H19 is upregulated in both oral cancer cell lines and cancer-associated fibroblasts (CAFs) and confers proliferation, migration, and glycolysis ability to oral CAFs [44]. Knock-down of H19 reduced tumor growth in nude mice [44]. The ELDR is over-expressed in OSCC patient samples and cell lines [34]. Intra-tumor injection of ELDR siRNA regressed OSCC cell line and patient-derived xenograft tumor growth in mice model indicating its therapeutic importance [34]. MALAT1 knock-down significantly increased cisplatin sensitivity and reduced OSCC xenograft tumor growth in nude mice [82]. The PCAT1 is upregulated in OSCC. Intratumor delivery of siRNA to PCAT1 significantly reduced xenograft tumor growth in nude mice [94]. TTN-AS1 is upregulated in OSCC. Knock-down of the lncRNA by shRNA significantly reduces xenograft tumor growth and inhibits the expression of Ki67 and PCNA in nude mice [107]. XIST is upregulated in human tongue cancer. CRISPR/Cas9 mediated knock-out of XIST reduced *in-vivo* tumor development in nude mice [113]. Phytochemical Cucurbitacin B (CuB), which is derived primarily from *Trichosanthes cucumerina* L. fruits, has the ability to inhibit *in-vivo* xenograft tumor growth by specifically inhibiting XIST, suggesting the therapeutic role of XIST [113]. On the other hand, FALEC is downregulated in tongue cancer and associated with a good prognosis [116]. Overexpression of FALEC reduced xenograft tumor growth, and Ki67 expression, and induced apoptosis in nude mice [116]. The MEG3 is downregulated in OSCC [123]. Overexpression of MEG3 significantly reduced xenograft tumor growth in nude mice. Further, MEG3 over-expressed tumors showed reduced vimentin expression, inactivation JAK–STAT pathway, and induction in TUNEL-positive apoptotic cells [123]. The therapeutic efficacy of these lncRNAs is summarized in Table 1. All these studies examine the effect of individual lncRNA in OSCC animal models. Although all these studies reported the promising therapeutic role of lncRNA in OSCC therapy, the combinatorial effects of these lncRNAs are not known. Further, bioavailability, toxicity, and organ specificity information are not clear. If these parameters are within acceptable limits, we can hypothesize that significantly top candidate lncRNA in individual OSCCs could be useful therapeutic molecules alone or in combination with traditional chemotherapeutic agents.

### Long non-coding RNA as circulatory biomarkers in oral cancer

**Saliva:** Saliva is the most readily obtained, least expensive, and non-invasive; it is therefore given careful consideration while creating biomarkers. Despite the fact that around 98% of the genome is non-protein coding, the amount of these genes in saliva is highly dubious because the majority of coding and non-coding RNA breaks down there [167]. A preliminary investigation utilizing saliva samples from patients with OSCC (*n* = 9) revealed the presence of MALAT-1 in every participant. However, the expression was not correlated with metastasis [168]. On the other hand, out of the 9 patients, 5 had HOTAIR detection; 3 of the 4 patients who had lymph node metastasis tested positive, which is greater than in the patients who did not have metastasis [168]. These findings suggest the importance of MALAT-1 and HOTAIR as possible diagnostic and prognostic biomarkers in saliva for OSCC. However, correlation analysis showed no association of the lncRNA biomarkers with age and gender [168]. Studies showed an association of HOTAIR with poor patient survival [168]. However, there was no information on specificity and sensitivity as salivary biomarkers. In tissue samples and cell lines, XIST is upregulated (Table 1). One study showed that loss of expression of XIST in the saliva samples had a significantly increased odds ratio in OSCC patients (OSCC: *n* = 59 *vs*. healthy control: *n* = 43), particularly in females [169]. The expression showed no correlation with alcohol consumption, betel quid chewing, or cigarette smoking habits. The ROC curve showed that the salivary XIST expression provides an acceptable discrimination accuracy to predict the risk of OSCC [169]. The lncRNAs present in the saliva samples are listed in Table 2.

**Table 2:** Clinical importance of circulatory long non-coding RNAs in OSCC

Name of lncRNA	Expression	Clinical importance	Ref.
**Saliva**			
**MALAT-1**	Up	Diagnostic and prognostic marker	[168,174]
**HOTAIR**			
**XIST**	Down	Prognostic importance, associated with increased risk of OSCC	[169]
**Blood (plasma and serum)**			
**CASC2**	Down	Prognosis and postoperative local recurrence	[170]
**PAPAS**	Up	Increased expression is associated with cancer stage and poor patient survival	[171]
**NCK1-AS1**	Up	Upregulated in early OSCC stage and promotes OSCC growth	[85]
**NR_1311012, ENST00000588803, NR_038323, and ENST00000412740**	Differential expression	Associated with OSCC early detection and staging	[172]
**AC007271.3**	Up	Combined expression of AC007271.3 with TSGF and SCCA showed significant sensitivity and specificity for the early diagnosis of OSCC	[175]
**LOC284454**	Up	It may have a good clinical diagnostic potential	[176]
**MAGI2-AS3 and CCDC144NL-AS1**	Up in serum exosomes	Increased expression may have diagnostic and prognostic importance in OSCC	[180]

**Blood:** Another sample source for OSCC liquid biopsy is peripheral blood samples (plasma or serum). The lncRNA CASC2 is down-regulated in OSCC tissue and plasma samples [170]. ROC curve analysis showed that the area under the curve is 0.8445 with a standard error of 0.03655 and a 95% confidence interval of 0.7728–0.9162. The down-regulation of CASC2 was significantly correlated with tumor size, but not age, gender, smoking, and drinking habits [170]. However, the patients (*n* = 122) with local recurrence had considerably higher plasma levels of CASC2 than patients without recurrence suggesting the importance of CASC2 in OSCC prognosis and postoperative local recurrence [170]. The lncRNA PAPAS promotes OSCC proliferation, invasion, and migration by increasing TGF-β1 expression [171]. Additionally, lncRNA PAPAS has been linked to poor patient survival and cancer stage, and its levels are elevated in plasma samples [171]. In contrast, NCK1-AS1 expression is higher in plasma samples from patients with early-stage OSCC (*n* = 55) compared to healthy controls, but not in patients with oral ulcers (*n* = 49) [85]. Overexpression of NCK1-AS1 is associated with decreased miR-100 expression and enhanced OSCC cell invasion and migration [85]. Furthermore, the expression levels of NCK1-AS1 do not significantly correlate with patient gender, age, tumor size, location, or TNM stage, according to the chi-squared test. The ROC curve analysis reveals that high NCK1-AS1 expression has diagnostic significance for OSCC. Another study with plasma samples from 67 OSCC patients, 16 oral premalignant lesions, and 19 healthy control subjects, identified differential expression of lncRNAs NR_1311012, ENST00000588803, NR_038323, and ENST00000412740 indicating their importance in early detection and OSCC staging [172]. The diagnostic effectiveness of the combined lncRNAs was more apparent than that of a single lncRNA, according to ROC curve and logistic regression analysis. Upregulation of HOXA11-AS, LINC00964, and MALAT-1 are observed in the plasma samples of 100 head and neck cancer patients indicating their importance in diagnosis [173]. Risk score analysis and ROC curve analysis showed their accuracy and specificity as biomarkers. However, no correlation with different clinical parameters like-age, sex, cancer stage, and lymph node metastasis was observed. Another study showed increased expression of MALAT-1 in TSCC (61 samples out of a total of 72 samples) and serum samples [174]. This high serum expression is significantly associated with cancer stages, distance tumor metastasis, and poor survival [174]. The ROC curve analysis indicates the sensitivity and specificity of MALAT1 as a tongue cancer biomarker [174]. Kaplan-Meier analysis showed high MALAT1 expression is associated with poor overall survival. Although the serum MALAT1 showed no significant correlations with gender, age, smoking, and alcohol consumption, it showed an association with distant tumor metastasis [174]. The lncRNA AC007271.3 is over-expressed in OSCC tissue and serum samples (*n* = 80) and is positively associated with clinical stage, lymphatic metastasis, and smoking history [175]. Further, combined expression of AC007271.3 with tumor-specific growth factor (TSGF), and squamous cell carcinoma antigen (SCCA) showed significant sensitivity and specificity as the novel circulating biomarkers for the early diagnosis of OSCC [175]. In comparison to the normal controls (*n* = 121), the serum of head and neck cancer patients (*n* = 212), including OSCC, showed a substantial upregulation of lncRNA LOC284454and had a good clinical diagnostic value determined by area under the ROC curve values of 0.931, 0.698, and 0.834, respectively [176]. However, there is no association found between the expression level of LOC284454 and clinical stages, gender, or age distribution. The plasma or serum lncRNAs are summarized in Table 2.

**Exosome:** Exosomes are 40–100 nm phospholipid-bound extracellular vesicles that induce therapy resistance, tumor immunity, angiogenesis, and the onset of cancer [177,178]. The lncRNAs in body fluids may reside in the extracellular vesicles or exosomes. The exosome-encapsulated lncRNAs are suggested to be well protected in circulation from RNAase-mediated degradation [179]. Dysregulation of lncRNAs in exosomes has recently been shown to alter the tumor microenvironment and cause cancer cells to exhibit an aggressive phenotype, metastasis, and therapy resistance [177]. For example, exosomal lncRNAs AFAP1-AS1, AGAP2-AS1, H19, HOTTIP, lincRNA-ROR, lncARSR, PART1, PCSEAT, RP11-838N2.4, SBF2-AS1, SNHG14, Sox2ot, TUC339, UCA1, and ZFAS1 are found to induce progression and chemoresistance of different cancers [177]. Despite the potential importance of exosomal lncRNAs in OSCC, their clinical and functional significance remains largely unexplored, prompting a further investigation in this area. The lncRNA MAGI2-AS3 and CCDC144NL-AS1 are significantly upregulated in the serum exosome samples from OSCC patients with or without lymph node metastasis suggesting their clinical importance [180] (Table 2). Further mechanistic investigation in OSCC cell lines showed that MAGI2-AS3 and CCDC144NL-AS1 induce cell proliferation, invasion, and migration by regulating the PI3K-AKT-mTOR pathway [180]. CCDC144NL-AS1 was strongly correlated with aging, and AC109587.1 and AC010978.1 were significantly correlated with the clinical stage.

### Long non-coding RNA in oral cancer clinical trial

Cancer-specific non-coding RNAs (ncRNAs) can potentially function as valuable biomarkers and therapeutic targets, enabling the tailoring of treatment plans to the unique needs of individual patients or patient subgroups. Recent studies indicate that non-coding RNAs hold significant promise for cancer diagnosis and treatment, either as standalone tools or in conjunction with established biomarkers. This is particularly promising because non-coding RNAs can simultaneously target a variety of druggable and non-druggable targets across multiple signaling pathways, and they exhibit tissue specificity and unique RNA characteristics. LncRNAs have been utilized as therapeutic and diagnostic biomarkers in several clinical trials. An observational trial is underway to evaluate the diagnostic and therapeutic potential of the MALAT-1 and its target miRNA-124 in saliva samples of OSCC patients (ClinicalTrials.gov Identifier: NCT05708209). The study has enrolled 40 participants, and the outcomes have not yet been published. Another study has examined the impact of the lncRNA DQ786243 on salivary expression of miRNA-146a and its potential utility for diagnosing oral malignant lesions in comparison to normal controls (NCT05730855). The study has been conducted on 45 enrolled participants, but the results and publications are not yet reported. A Phase II multicenter clinical study with 104 enrolled participants is currently underway to assess the therapeutic efficacy of the drug Dacomitinib on EGFR-associated advanced oral cancer with low expression of the lncRNA EGFR-AS1 (NCT04946968). The low expression of EGFR-AS1 is determined to be a companion diagnostic biomarker of oral cancer, and the findings of this study may highlight the significance of EGFR-AS1 as a therapeutic and diagnostic tool for treating oral cancer. Overall, these ongoing clinical investigations underscore the growing interest and potential of cancer-specific non-coding RNAs as biomarkers and therapeutic targets to personalize cancer management.

### Challenges and future perspective

The lncRNA has significant potential to advance the understanding of OSCC and inform novel strategies for prevention, diagnosis, and treatment. Despite this promise, considerable uncertainties persist, and further research is necessary to fully elucidate the complex roles of lncRNAs in OSCC. (1) Unexplored lncRNAs: Many lncRNAs have not yet been thoroughly investigated, and their functions in OSCC remain largely unknown. (2) Pre-clinical and clinical research: To fully harness the potential of lncRNAs in OSCC, further research is required in both pre-clinical and clinical settings. This includes refining *in-vivo* stability and organ-specific targeting, thorough validation, and follow-up studies to ensure the effective translation of lncRNA-based therapies. (3) Tumor microenvironment and immune system: It is necessary to assess the function of lncRNAs in the tumor microenvironment, particularly in regulating the immune system and resistance to therapy. (4) Animal models: There is a dearth of humanized *in-vivo* animal models to understand the role of lncRNAs in the development and progression of OSCC. (5) Early detection and disease progression: Little is known about the function of lncRNAs in the progression of OSCC from early dysplastic lesions to fully developed tumors. (6) Population level variability: to comprehensively understand the complex interplay of factors influencing OSCC, extensive mechanistic research is needed on patient cohort studies from diverse global locations, considering the effects of age, sex, behavioral risk factors, genetic predisposition, microbial infection, and poor diet on OSCC. (7) Clinical and functional significance of lncRNAs: Investigation is required on the clinical and functional significance of lncRNAs in bodily fluids and exosomes, which could lead to the development of faster and more accurate testing kits for OSCC. The insights gained from addressing these challenges will lay the groundwork for substantial breakthroughs in healthcare, prognosis, and therapeutic options for the management of OSCC in the near future.

## Conclusion

In summary, this review outlines the substantial role that lncRNAs play in the development, diagnosis, and treatment of OSCC. Current diagnostic and therapeutic biomarkers for OSCC are insufficient as primary diagnostic tools, but recent research highlights the importance of lncRNAs in OSCC progression, diagnosis, and treatment. Numerous lncRNAs are upregulated in OSCC, such as ANRIL, HOTAIR, MALAT-1, NEAT1, PVT1, and XIST, while others like FALEC, GAS5, and MEG3 are down-regulated. These lncRNAs interact with DNA, RNA, proteins, or miRNAs to regulate various cellular processes crucial in OSCC, including cell survival, proliferation, invasion, metastasis, immune system, and drug resistance. Some lncRNAs like MALAT-1, HOTAIR, and XIST show potential clinical significance in OSCC diagnosis and prognosis. Clinical trials are ongoing for lncRNAs like MALAT-1, DQ786243, and EGFR-AS1. Further research with larger patient samples is essential to fully comprehend the implications of lncRNAs in OSCC management, potentially leading to more accurate diagnostic tools and effective therapeutic strategies for this challenging disease.

## Data Availability

None.

## Abbreviations

ABCB5: ATP Binding Cassette Subfamily B Member 5
ATGL: Adipose Triglyceride Lipase
Bax: Bcl-2-Associated X Protein
Bcl-2: B-Cell Lymphoma-2
CCR7: C-C Chemokine Receptor 7
CHSY1: Chondroitin Sulfate Synthase 1
circRNA: Circular RNA
CLDN4: Claudin 4
CXCR4/7: CXC Chemokine Receptor 4/7
EGFR: Epidermal Growth Factor Receptor
EMT: Epithelial-Mesenchymal Transition
EZH2: Enhancer of Zeste Homolog 2
FDA: The United States Food and Drug Administration
FGF7: Fibroblast Growth Factor 7
PFKFB3: Fructose-2,6-Biphosphatase 3
FZD4: Frizzled Class Receptor 4
GATA3: GATA Binding Protein 3
GLUT-1: Glucose Transporter 1 (Solute Carrier Family 2, Facilitated Glucose Transporter Member 1)
HCC: Hepatocellular Carcinoma
HNSCC: Head and Neck Squamous Cell Carcinomas
HK2: Hexokinase 2
HIF-1α: Hypoxia-Inducible Factor (HIF)-1alpha subunit
HNRNPC: Heterogeneous Nuclear Ribonucleoprotein C
HPV: Human Papillomavirus
ILF3: Interleukin Enhancer-Binding Factor 3
JAG: Jagged Canonical Notch Ligand 1
KDM5A: Lysine-Specific Demethylase 5A
LC3B: Microtubule Associated Protein 1 Light Chain 3 beta
LncRNA: Long Non-Coding RNA
MAPK: Mitogen-Activated Protein Kinase
MMP: Matrix Metalloproteinases
MDR1: Multidrug Resistance 1
miRNA: MicroRNA
mTOR: Mammalian Target of Rapamycin
MDSCs: Myeloid Inhibitory Cells
NK-cells: Natural Killer cells
NF-κB: Nuclear Factor Kappa-Light-Chain-Enhancer of Activated B Cells
NcRNA: Non-coding RNA
OPMD: Oral Potentially Malignant Disorder
OSCC: Oral Squamous Cell Carcinomas
p16INK4a: CDK4 Inhibitor P16-INK4 (Cyclin Dependent Kinase Inhibitor 2A)
PAM50: Prediction Analysis of Microarray 50
PI3K: Phosphoinositide 3-Kinase
piRNA: PIWI-interacting RNA
PAI-1: Plasminogen Activator Inhibitor-1
PRC2: Polycomb Repressive Complex 2
PTEN: Phosphatase and Tensin Homolog
PKM2: Pyruvate kinase 2; Tregs: Regulatory T cells
snoRNA: Small Nucleolar RNA
SOCS5/6: Suppressor of Cytokine Signaling 5
SRSF1: Serine and Arginine-Rich Splicing Factor 1
STAT3: Signal Transducer and Activator of Transcription 3
TCF7L2: Transcription Factor-7-Like 2
TGFβ-1: Transforming Growth Factor-beta1
TME: Tumor Microenvironment; TSCC: Tongue Squamous Cell Carcinoma
VEGFR: Vascular Endothelial Growth Factor Receptor
VHL: Von Hippel-Lindau Tumor Suppressor
YAP1: Yes-Associated Protein 1
